# Supplementary material for: Omega-3 Fatty Acids for Depression in the Elderly and Patients with Dementia: A Systematic Review and Meta-Analysis
Source: Healthcare (Basel). 2024 Feb 23;12(5):536. doi: 10.3390/healthcare12050536 (PMC10931076; doi:10.3390/healthcare12050536)
Supplement: Supplementary file 1 [file healthcare-12-00536-s001.zip › healthcare-2833510-supplementary.pdf]

Table S1. **PRISMA Checklist**

Section and Topic	Item	Checklist item	Location where item is reported
**TITLE**			
Title	1	Identify the report as a systematic review.	Title
**ABSTRACT**			
Abstract	2	See the PRISMA 2020 for Abstracts checklist.	Abstract
**INTRODUCTION**			
Rationale	3	Describe the rationale for the review in the context of existing knowledge.	Introduction
Objectives	4	Provide an explicit statement of the objective(s) or question(s) the review addresses.	Introduction
**METHODS**			
Eligibility criteria	5	Specify the inclusion and exclusion criteria for the review and how studies were grouped for the syntheses.	Methods
Information sources	6	Specify all databases, registers, websites, organisations, reference lists and other sources searched or consulted to identify studies. Specify the date when each source was last searched or consulted.	Methods
Search strategy	7	Present the full search strategies for all databases, registers and websites, including any filters and limits used.	Methods, Table S2
Selection process	8	Specify the methods used to decide whether a study met the inclusion criteria of the review, including how many reviewers screened each record and each report retrieved, whether they worked independently, and if applicable, details of automation tools used in the process.	Methods

Data collection process	9	Specify the methods used to collect data from reports, including how many reviewers collected data from each report, whether they worked independently, any processes for obtaining or confirming data from study investigators, and if applicable, details of automation tools used in the process.	Methods
Data items	10a	List and define all outcomes for which data were sought. Specify whether all results that were compatible with each outcome domain in each study were sought (e.g. for all measures, time points, analyses), and if not, the methods used to decide which results to collect.	Methods
	10b	List and define all other variables for which data were sought (e.g. participant and intervention characteristics, funding sources). Describe any assumptions made about any missing or unclear information.	Methods, Table 1, Table 2
Study risk of bias assessment	11	Specify the methods used to assess risk of bias in the included studies, including details of the tool(s) used, how many reviewers assessed each study and whether they worked independently, and if applicable, details of automation tools used in the process.	Methods, Table S4a, Table S4b
Effect measures	12	Specify for each outcome the effect measure(s) (e.g. risk ratio, mean difference) used in the synthesis or presentation of results.	Methods
Synthesis methods	13a	Describe the processes used to decide which studies were eligible for each synthesis (e.g. tabulating the study intervention characteristics and comparing against the planned groups for each synthesis (item #5)).	Methods, Figure 1, Table 1-2, Table S3
	13b	Describe any methods required to prepare the data for presentation or synthesis, such as handling of missing summary statistics, or data conversions.	Methods
	13c	Describe any methods used to tabulate or visually display results of individual studies and syntheses.	Methods

	13d	Describe any methods used to synthesize results and provide a rationale for the choice(s). If meta-analysis was performed, describe the model(s), method(s) to identify the presence and extent of statistical heterogeneity, and software package(s) used.	Methods
	13e	Describe any methods used to explore possible causes of heterogeneity among study results (e.g. subgroup analysis, meta-regression).	Methods
	13f	Describe any sensitivity analyses conducted to assess robustness of the synthesized results.	Methods
Reporting bias assessment	14	Describe any methods used to assess risk of bias due to missing results in a synthesis (arising from reporting biases).	Methods, Figure 2
Certainty assessment	15	Describe any methods used to assess certainty (or confidence) in the body of evidence for an outcome.	Methods
**RESULTS**			
Study selection	16a	Describe the results of the search and selection process, from the number of records identified in the search to the number of studies included in the review, ideally using a flow diagram.	Results, Figure 1, Table S2-S3
	16b	Cite studies that might appear to meet the inclusion criteria, but which were excluded, and explain why they were excluded.	Results, Table S3
Study characteristics	17	Cite each included study and present its characteristics.	Results, Table 1
Risk of bias in studies	18	Present assessments of risk of bias for each included study.	Results, Table S4a-b
Results of individual studies	19	For all outcomes, present, for each study: (a) summary statistics for each group (where appropriate) and (b) an effect estimate and its precision (e.g. confidence/credible interval), ideally using structured tables or plots.	Results, Figure S1, Table S4a-b
Results of syntheses	20a	For each synthesis, briefly summarise the characteristics and risk of bias among contributing studies.	Results, Table S4a-b

	20b	Present results of all statistical syntheses conducted. If meta-analysis was done, present for each the summary estimate and its precision (e.g. confidence/credible interval) and measures of statistical heterogeneity. If comparing groups, describe the direction of the effect.	Results, Figure 2
	20c	Present results of all investigations of possible causes of heterogeneity among study results.	Results, Table 2
	20d	Present results of all sensitivity analyses conducted to assess the robustness of the synthesized results.	NA
Reporting biases	21	Present assessments of risk of bias due to missing results (arising from reporting biases) for each synthesis assessed.	Results, Table S4a-b
Certainty of evidence	22	Present assessments of certainty (or confidence) in the body of evidence for each outcome assessed.	Results, Figure S2
**DISCUSSION**			
Discussion	23a	Provide a general interpretation of the results in the context of other evidence.	Discussion
	23b	Discuss any limitations of the evidence included in the review.	Discussion
	23c	Discuss any limitations of the review processes used.	Discussion
	23d	Discuss implications of the results for practice, policy, and future research.	Discussion
**OTHER INFORMATION**			
Registration and protocol	24a	Provide registration information for the review, including register name and registration number, or state that the review was not registered.	Methods
	24b	Indicate where the review protocol can be accessed, or state that a protocol was not prepared.	Methods, Table S2-S3
	24c	Describe and explain any amendments to information provided at registration or in the protocol.	Methods, Table S2-S3

Support	25	Describe sources of financial or non-financial support for the review, and the role of the funders or sponsors in the review.	NA
Competing interests	26	Declare any competing interests of review authors.	NA
Availability of data, code and other materials	27	Report which of the following are publicly available and where they can be found: template data collection forms; data extracted from included studies; data used for all analyses; analytic code; any other materials used in the review.	Results, Table S2-S4

From: Page MJ, McKenzie JE, Bossuyt PM, Boutron I, Hoffmann TC, Mulrow CD, et al. The PRISMA 2020 statement: an updated guideline for reporting systematic reviews. BMJ 2021;372:n71. doi: 10.1136/bmj.n71

Table S2 - Keywords and search results in different databases

			**1100**
Database	Keyword	Date	Results
PubMed	('omega-3 fatty acids' OR 'fish oil' OR 'docosahexaenoic acid' OR 'eicosapentaenoic acid' OR 'dha' OR 'epa') AND ('depression' OR 'depressive') AND ('dementia' OR 'alzheimer')	2024.01.24	177
Embase	('omega-3 fatty acids' OR 'fish oil' OR 'docosahexaenoic acid' OR 'eicosapentaenoic acid' OR 'dha' OR 'epa') AND ('depression' OR 'depressive') AND ('dementia' OR 'alzheimer')	2024.01.24	163
Cochrane CENTRAL	('omega-3 fatty acids' OR 'fish oil' OR 'docosahexaenoic acid' OR 'eicosapentaenoic acid' OR 'dha' OR 'epa') AND ('depression' OR 'depressive') AND ('dementia' OR 'alzheimer')	2024.01.24	52
Web of Science	('omega-3 fatty acids' OR 'fish oil' OR 'docosahexaenoic acid' OR 'eicosapentaenoic acid' OR 'dha' OR 'epa') AND ('depression' OR 'depressive') AND ('dementia' OR 'alzheimer')	2024.01.24	207
			599

Database	Keyword	Date	Results
PubMed	('omega-3 fatty acids' OR 'fish oil' OR 'docosahexaenoic acid' OR 'eicosapentaenoic acid' OR 'dha' OR 'epa') AND ('depression' OR 'depressive') AND ('dementia' OR 'alzheimer') AND ('random' OR 'randomized' OR 'randomised')	2024.01.24	39
Embase	('omega-3 fatty acids' OR 'fish oil' OR 'docosahexaenoic acid' OR 'eicosapentaenoic acid' OR 'dha' OR 'epa') AND ('depression' OR 'depressive') AND ('dementia' OR 'alzheimer') AND ('random' OR 'randomized' OR 'randomised')	2024.01.24	81
Cochrane CENTRAL	('omega-3 fatty acids' OR 'fish oil' OR 'docosahexaenoic acid' OR 'eicosapentaenoic acid' OR 'dha' OR 'epa') AND ('depression' OR 'depressive') AND ('dementia' OR	2024.01.24	37

	'alzheimer') AND ('random' OR 'randomized' OR 'randomised')		
Web of Science	('omega-3 fatty acids' OR 'fish oil' OR 'docosahexaenoic acid' OR 'eicosapentaenoic acid' OR 'dha' OR 'epa') AND ('depression' OR 'depressive') AND ('dementia' OR 'alzheimer') AND ('random' OR 'randomized' OR 'randomised')	2024.01.24	46
			203

Database	Keyword	Date	Results
PubMed	('docosahexaenoic acid' OR 'eicosapentaenoic acid' OR 'dha' OR 'epa') AND ('depression' OR 'depressive') AND ('dementia' OR 'alzheimer') AND ('random' OR 'randomized' OR 'randomised')	2024.01.24	23
Embase	('docosahexaenoic acid' OR 'eicosapentaenoic acid' OR 'dha' OR 'epa') AND ('depression' OR 'depressive') AND ('dementia' OR 'alzheimer') AND ('random' OR 'randomized' OR 'randomised')	2024.01.24	88
Cochrane CENTRAL	('docosahexaenoic acid' OR 'eicosapentaenoic acid' OR 'dha' OR 'epa') AND ('depression' OR 'depressive') AND ('dementia' OR 'alzheimer') AND ('random' OR 'randomized' OR 'randomised')	2024.01.24	25
Web of Science	('docosahexaenoic acid' OR 'eicosapentaenoic acid' OR 'dha' OR 'epa') AND ('depression' OR 'depressive') AND ('dementia' OR 'alzheimer') AND ('random' OR 'randomized' OR 'randomised')	2024.01.24	39
			175

Database	Keyword	Date	Results
PubMed	('omega-3 fatty acids' OR 'fish oil' OR 'dha' OR 'epa') AND ('depression' OR 'depressive') AND ('mci' OR 'mild cognitive impairment') AND ('random' OR 'randomized' OR 'randomised')	2024.01.24	41
Embase	('omega-3 fatty acids' OR 'fish oil' OR 'dha' OR 'epa') AND ('depression' OR 'depressive') AND ('mci' OR 'mild	2024.01.24	19

	cognitive impairment') AND ('random' OR 'randomized' OR 'randomised')		
Cochrane CENTRAL	('omega-3 fatty acids' OR 'fish oil' OR 'dha' OR 'epa') AND ('depression' OR 'depressive') AND ('mci' OR 'mild cognitive impairment') AND ('random' OR 'randomized' OR 'randomised')	2024.01.24	13
Web of Science	('omega-3 fatty acids' OR 'fish oil' OR 'dha' OR 'epa') AND ('depression' OR 'depressive') AND ('mci' OR 'mild cognitive impairment') AND ('random' OR 'randomized' OR 'randomised')	2024.01.24	24
			97

Database	Keyword	Date	Results
PubMed	('omega-3 fatty acids' OR 'fish oil' OR 'dha' OR 'epa') AND ('depression' OR 'depressive') AND ('memory loss') AND ('random' OR 'randomized' OR 'randomised')	2024.01.24	16
Embase	('omega-3 fatty acids' OR 'fish oil' OR 'dha' OR 'epa') AND ('depression' OR 'depressive') AND ('memory loss') AND ('random' OR 'randomized' OR 'randomised')	2024.01.24	6
Cochrane CENTRAL	('omega-3 fatty acids' OR 'fish oil' OR 'dha' OR 'epa') AND ('depression' OR 'depressive') AND ('memory loss') AND ('random' OR 'randomized' OR 'randomised')	2024.01.24	4
Web of Science	('omega-3 fatty acids' OR 'fish oil' OR 'dha' OR 'epa') AND ('depression' OR 'depressive') AND ('memory loss') AND ('random' OR 'randomized' OR 'randomised')	2024.01.24	0
			26

Table S3 Excluded studies and reasons

No.	First Author	Publication Year	Title	Journal/Book	Exclusion reasons
1	Borsini	2021	Omega-3 polyunsaturated fatty acids protect against inflammation through production of LOX and CYP450 lipid mediators: relevance for major depression and for human hippocampal neurogenesis	Molecular Psychiatry	No relevant outcome data
2	Ibrahim-Fouad	2020	Combination of Omega 3 and Coenzyme Q10 Exerts Neuroprotective Potential Against Hypercholesterolemia-Induced Alzheimer's-Like Disease in Rats	Neurochemical Research	No relevant outcome data
3	Patnode	2020	Screening for Cognitive Impairment in Older Adults: An Evidence Update for the U.S. Preventive Services Task Force	Screening for Cognitive Impairment in Older Adults: An Evidence Update for the U.S. Preventive Services Task Force	No relevant outcome data
4	Fougère	2018	Red blood cell membrane omega-3 fatty acid levels and physical performance: Cross-sectional data from the MAPT study	Nutrition Journal - Clinical Nutrition	Incomplete data
5	Fougère	2017	Omega-3 fatty acid levels in red blood cell membranes and physical decline over 3 years: longitudinal data from the MAPT study	GeroScience	No relevant outcome data
6	Diniz	2016	Circulating biosignatures of late-life depression (LLD): Towards a	Journal of Psychiatric Research	Incomplete data

			comprehensive, data-driven approach to understanding LLD pathophysiology		
7	Schuchardt	2016	Genetic Variants of the FADS Gene Cluster Are Associated with Erythrocyte Membrane LC PUFA Levels in Patients with Mild Cognitive Impairment	The journal of nutrition, health & aging	Incomplete data
8	Burckhardt	2016	Omega-3 fatty acids for the treatment of dementia	Cochrane Database of Systematic Reviews	Review
9	Cardoso	2016	Dietary DHA and health: cognitive function ageing	Nutrition Research Reviews	Review
10	Milte	2011	Erythrocyte polyunsaturated fatty acid status, memory, cognition and mood in older adults with mild cognitive impairment and healthy controls	Prostaglandins, Leukotrienes and Essential Fatty Acids	Incomplete data
11	Seppi	2011	The Movement Disorder Society Evidence-Based Medicine Review Update: Treatments for the non-motor symptoms of Parkinson's disease	Movement Disorders	Review
12	Sinn	2010	Oiling the brain: a review of randomized controlled trials of omega-3 fatty acids in psychopathology across the lifespan	Nutrients	Review
13	Solfrizzi	2008	Dietary fatty acids, age-related cognitive decline, and mild cognitive impairment	The journal of nutrition, health & aging	Review
14	Kidd	2007	Omega-3 DHA and EPA for cognition, behavior, and mood: clinical findings and	Alternative Medicine Review	Letter

			structural-functional synergies with cell membrane phospholipids		
15	Assisi	2006	Fish oil and mental health: the role of n-3 long-chain polyunsaturated fatty acids in cognitive development and neurological disorders	International Clinical Psychopharmacology - Lippincott	Review
16	Freeman	2006	Omega-3 fatty acids: evidence basis for treatment and future research in psychiatry	Journal of Clinical Psychiatry	Review
17	Fugh-Berman	1999	Dietary supplements and natural products as psychotherapeutic agents	Psychosomatic Medicine	Review

Table S4a - Risk of bias assessment

First author & Year	Randomization process	Intervention adherence	Missing outcome data	Outcome measurement	Selective reporting	Overall RoB
Freund-Levi et al., 2008	L	S2	L	L	L	L
Freund-Levi et al., 2014	L	L3	L	L	L	L
Giudici et al., 2020	L	L3	L	L	L	L
Hashimoto et al., 2017	L	L3	L	L	L	L
Lin et al., 2022	L	L3	L	L	L	L
Maltais et al., 2019	L	L3	L	L	L	L
Rondanelli et al., 2012	L	L3	L	L	L	L
Sinn et al., 2012	L	L3	S	L	L	L
van de Rest et al., 2009	L	L3	L	L	L	L

1. The study employed a waitlist control group design, which resulted in a more balanced comparison among different groups.
2. The differences in protocols among various groups may affect adherence and outcome.
3. Both groups were randomized to receive exercise interventions, and the study design utilized a balanced protocol, which minimized the impact on adherence.

H, high risk of bias; L, low risk of bias; S, some risk of bias.

Table S4b - Detailed quality assessment of included studies using Cochrane risk of bias 2 tool

First author & Year	Randomization process	Intervention adherence	Missing outcome data	Outcome measurement	Selective reporting	Overall RoB
	L	S2	L	L	L	L
Freund-Levi et al., 2008	The study employed a randomized, double-blind, placebo-controlled design with sealed envelopes, ensuring appropriate randomization and allocation concealment.	The study monitored adherence through treatment protocols but did not detail specific measures to enhance adherence. Possible factors affecting adherence include gastrointestinal issues and capsule size, leading to dropouts.	The analysis accounted for dropouts and used per-protocol analysis, minimizing the bias from missing data, although the reasons for dropouts were noted.	Outcomes were assessed using standardized and reliable tools (NPI, MADRS), and the study design ensured that the outcome assessors were blinded to the intervention.	The study was registered (ClinicalTrials.gov identifier: NCT00211159), and outcomes reported appear comprehensive, with no evidence of selective outcome reporting.	Given the detailed methodological information and adherence to a rigorous study design, the study exhibits a low overall risk of bias.
	L	L3	L	L	L	L
Freund-Levi et al., 2014	The study was a randomized, double-blind, placebo-controlled trial ensuring appropriate randomization and allocation concealment.	Adherence to intervention was monitored, with specific dosages of DHA and EPA provided and adherence likely encouraged by study	The study accounted for dropouts and protocol violations. Missing data and its impact on the study outcome	Outcome measures were objective, utilizing biochemical assays for oxidative stress and inflammation markers, with	The study registered its protocol (ClinicalTrials.gov Identifier: NCT0021115) and reported outcomes comprehensively,	The study demonstrates a low overall risk of bias with a well-conducted randomization process, adherence monitoring, comprehensive

	No evidence of a breach in randomization process.	protocols. No specific factors affecting adherence were detailed, thus assumed controlled.	appear to have been managed adequately.	blinded assessment implied by the study's double-blind design.	with no indication of selective reporting.	outcome measurement, and transparency in reporting.
	L	L3	L	L	L	L
Giudici et al., 2020	The study used a computer-generated randomization procedure with block sizes and stratification by center, ensuring appropriate randomization and allocation concealment.	Adherence was monitored through session attendance and capsule counts, with participants considered adherent if they met predefined thresholds.	The study provided details on handling missing data and performed analyses on a modified intention-to-treat basis, minimizing bias.	Outcome measures were objective, using standardized assessments for intrinsic capacity domains, and assessors were blinded to group assignments.	The study outcomes are comprehensively reported, with no evidence suggesting selective reporting of results.	The study demonstrates a low overall risk of bias with a rigorous methodology, ensuring the reliability of its findings.
	L	L3	L	L	L	L
Hashimoto et al., 2017	The study utilized a randomized, double-blind, placebo-controlled design, ensuring appropriate allocation and concealment procedures.	High adherence was suggested by significant differences in EPA levels and the EPA/AA ratio in the active group versus placebo at 12 months, indicating participants followed the intervention protocol effectively. This corresponds to attribute 3: Both groups were	The study accounted for dropouts and conducted a complete analysis on participants who completed the study, reducing potential bias from missing data.	Objective measures were used for cognitive and mental health assessments, and it's implied that outcome assessors were blinded to intervention groups.	There's no indication of selective reporting; the study appears to have reported on all expected outcomes comprehensively.	Considering the robust design and execution, the study shows a low overall risk of bias, supporting the reliability of its findings on the benefits of DHA-enriched meals for cognitive function and mental

		randomized to receive dietary interventions, and the study design utilized a balanced protocol, minimizing the impact on adherence.				health in elderly populations.
	L	L3	L	L	L	L
Lin et al., 2022	The study employed a randomized, double-blind, placebo-controlled design, ensuring appropriate randomization and allocation concealment.	Adherence was monitored with scheduled clinical visits, blood testing, and pill counts, indicating a structured approach to measure and maintain adherence.	The study accounted for dropouts and conducted intention-to-treat analyses, reducing the risk of bias from missing data.	Outcome measures were objective and likely assessed blindly, using standardized tools for cognitive, functional, and mood status assessments.	The study outcomes are reported comprehensively, with no evidence suggesting selective reporting of results.	The study demonstrates a low overall risk of bias with rigorous methodology, ensuring the reliability of its findings on the impact of omega-3 fatty acids.
	L	L3	L	L	L	L
Maltais et al., 2019	Utilized computer-generated randomization with block sizes and stratification by center, ensuring proper allocation concealment.	Adherence measured by session attendance and capsule counts. No direct categorization attribute provided, but detailed monitoring indicates a structured approach to adherence.	Managed missing data effectively, with analyses likely accounting for dropouts.	Used the GDS-15 scale for objective measurement of depressive symptoms, implying blinded assessment.	No evidence of selective reporting. Comprehensive analysis of depressive symptoms across different severities.	Low overall risk of bias suggested by thorough methodology and adherence to protocol, contributing valuable insights into the effects of lifestyle and omega-3 interventions on depressive symptoms.

	L	L3	L	L	L	L
Rondanelli et al., 2012	The study utilized a randomized, double-blind, placebo-controlled design, indicating a low risk of bias in randomization.	Adherence was assessed through capsule counts, suggesting good compliance and monitoring, with no adverse effects reported.	The study provided clear reporting on the handling of missing data, indicating a structured approach to minimize bias.	Outcomes were assessed using standardized tools, implying objective and consistent measurement, with blinded assessors.	The study appears to report comprehensively on its findings, with no evidence of selective reporting detected.	Given the study's methodological strengths, it demonstrates a low overall risk of bias, enhancing the reliability of its conclusions on cognitive and nutritional improvements.
	L	L3	S	L	L	L
Sinn et al., 2012	The study utilized a randomized, double-blind, placebo-controlled design with minimization for allocation, indicating an appropriate randomization process.	High adherence was reported with capsule counts and adverse events monitored, indicating good compliance.	The study addressed missing data adequately, although the approach for handling missing data could potentially introduce bias if not all missing data were accounted for systematically.	Objective measures were used for depressive symptoms, cognitive functions, and quality of life assessments, with indications that outcome assessors were blinded.	No evidence of selective reporting was found, and the study seems to report comprehensively on its findings.	Given the robust methodology and adherence to protocol, the study demonstrates a low overall risk of bias, supporting the reliability of its conclusions regarding the beneficial effects of DHA and EPA on mental health in elderly people with MCI.

van de Rest et al., 2009	L	L3	L	L	L	L
	Employed a randomized, double-blind, placebo-controlled trial design, indicating appropriate randomization and allocation concealment methods.	High adherence to the intervention was confirmed by capsule counts and plasma EPA-DHA concentration changes, indicating effective monitoring and participant compliance.	The study handled missing data appropriately, with a low dropout rate and analyses that included most participants, reducing potential bias.	Quality of life was measured using a standardized instrument (WHOQOL-BREF), and the double-blind design ensured outcome assessors were blinded to intervention allocation.	No evidence of selective reporting. The study reported on its primary and secondary outcomes comprehensively.	Demonstrating a low overall risk of bias due to its methodological rigor, supporting the reliability of its findings on the lack of impact of fish oil supplementation on quality of life in the study population.

Table S5. GRADE Summary of findings for the use of Omeg-3 acid to improve depression in patients with dementia.

**Analyses**	***k***	**Effect size(SMD)**	**95% CIs**	***I^2***	***Quality of evidence***
Omeg-3 acid	5	-0.147	-0.324 to 0.049	0.00%	⊕⊕⊕○ Moderate quality, Some risk of bias in design; Inconsistency
Length	16	-0.170	-0.298 to -0.042	0.00%	⊕⊕⊕⊕ Indicates high confidence in the current evidence-based effect estimates.
Cognition	16	-0.151	-0.266 to 0.010	0.00%	⊕⊕⊕⊕ Indicates high confidence in the current evidence-based effect estimates.
DHA/ Dosage	13	-0.177	-0.317 to -0.038	14.04%	⊕⊕⊕○ Moderate quality, Needed to determine the optimal dosage.
EPA/ Dosage	11	-0.156	-0.314 to 0.002	18.38%	⊕⊕⊕○ Moderate quality, Needed to determine the optimal dosage.

Abbreviations: GRADE: Grading of Recommendations, Assessment, Development, and Evaluations; ES: Effect size for SMD; SMD: standardized mean difference; k: number of arms in studies; DHA: docosahexaenoic acid; EPA: eicosapentaenoic acid; CI: cognitive impairment. ⊕⊕⊕⊕: High quality; ⊕⊕⊕○: Moderate quality; ⊕⊕○○: Low quality; ⊕○○○: Very low quality

Table S6. List of Studies Eligible for Subgroup Analysis (Meta-Analysis)

Study	*Freund-Levi et al., 2008*	*Hashimoto et al., 2017*	*Lin et al., 2022*	*Rondanelli et al., 2012*	*Sinn et al., 2012*
Intervention type					
EPA			✓		✓
DHA		✓	✓		✓
DHA+EPA	✓		✓		
DHA+OS				✓	
Length					
3m				✓	
6m	✓	✓	✓		✓
12m	✓	✓	✓		
24m			✓		
Diagnosis					
MCI-Mild	✓		✓	✓	✓
Moderate	✓	✓	✓	✓	✓
Severe		✓			
DHA/ Dosage (Day)					
High	✓	✓			✓
Moderate			✓	✓	
Low			✓		✓
EPA/ Dosage (Day)					
High			✓		✓
Moderate	✓		✓		
Low				✓	✓
Rating scale					
GDS			✓	✓	✓
non-GDS	✓	✓			

Abbreviations: SMD: standardized mean difference; k: number studies; DHA: docosahexaenoic acid; EPA: eicosapentaenoic acid; OS: other supplements; AD: Alzheimer's disease; MCI: mild cognitive impairment; CI: cognitive impairment; GDS: geriatric depression scale.

Figure S1. Summary of ROB

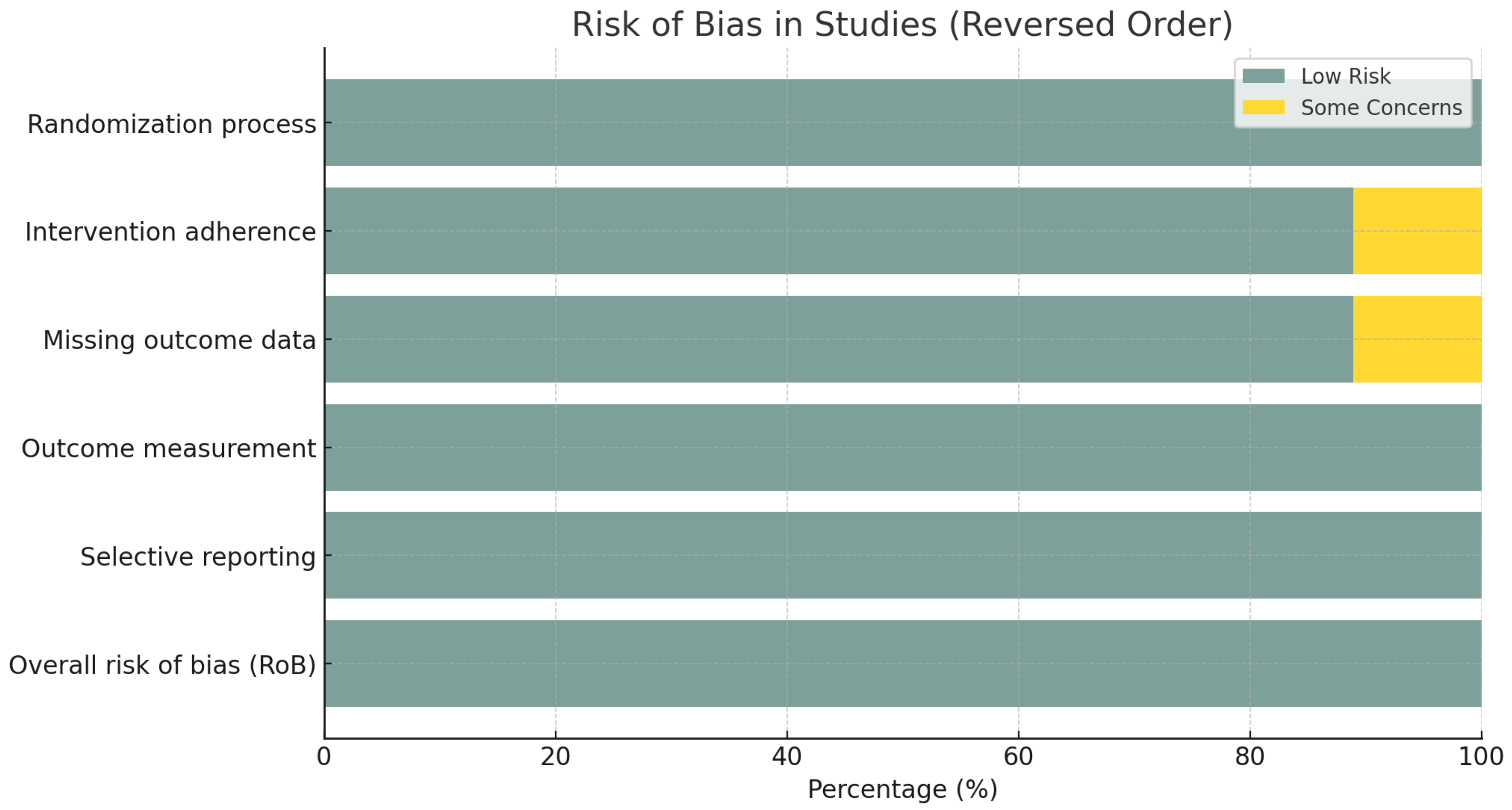

Figure S2. Summary of ROB

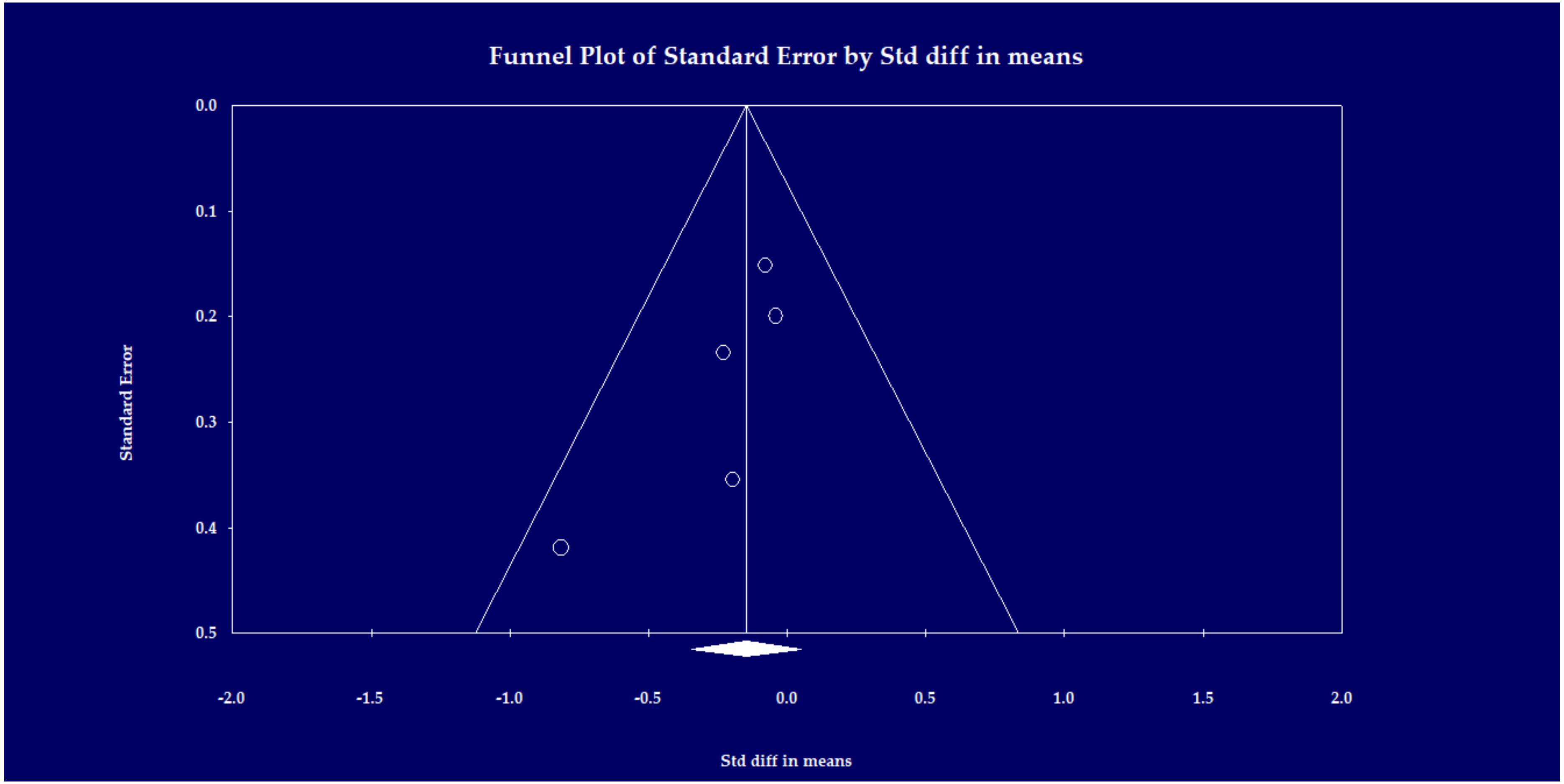

Egger's Test of the Intercept. The results showed an intercept (B0) of -1.90, with a 95% confidence interval ranging from -4.43 to 0.64. The 1-tailed p-value is 0.05, and the 2-tailed p-value is 0.10.
